# Implementierung und Evaluation einer Telefonhotline zur professionellen Ersthilfe bei psychischen Belastungen durch die COVID-19-Pandemie in Baden-Württemberg

**DOI:** 10.1007/s00115-021-01089-x

**Published:** 2021-03-16

**Authors:** Ruben Vonderlin, Miriam Biermann, Michael Konrad, Martin Klett, Nikolaus Kleindienst, Josef Bailer, Stefanie Lis, Martin Bohus

**Affiliations:** 1 Institut für Psychiatrische und Psychosomatische Psychotherapie, Zentralinstitut für Seelische Gesundheit, Medizinische Fakultät Mannheim, Universität Heidelberg, J5, 68159 Mannheim, Deutschland; 2 Ministerium für Soziales und Integration Baden-Württemberg, Stuttgart, Deutschland; 3 Landespsychotherapeutenkammer Baden-Württemberg, Stuttgart, Deutschland; 4 Abteilung für Klinische Psychologie, Zentralinstitut für Seelische Gesundheit, Medizinische Fakultät Mannheim, Universität Heidelberg, Mannheim, Deutschland; 5 McLean Hospital, Harvard Medical School, Boston, MA USA

**Keywords:** Coronavirus, COVID-19 Pandemie, Hotline, Psychologische Hilfe, Psychische Gesundheit, Coronavirus, COVID-19 pandemic, Hotline, Psychological counseling, Mental health

## Abstract

**Hintergrund:**

Die COVID-19-Pandemie stellt für viele Menschen eine deutliche psychische Belastung dar, für deren Bewältigung gerade während der ersten Welle der Pandemie sofort verfügbare professionelle Ansprechpartner fehlten.

**Ziel der Arbeit:**

In Baden-Württemberg wurde unter Federführung des Ministeriums für Soziales und Integration im April 2020 eine Telefonhotline zur psychologischen Ersthilfe bei Corona-assoziierten Problemen implementiert, für die gesamte Bevölkerung geöffnet und evaluiert.

**Material und Methoden:**

Im Zeitraum vom 22.04. bis zum 24.07.2020 nahmen 753 ehrenamtliche, psychotherapeutisch ausgebildete Berater*innen unterschiedlicher Berufsgruppen insgesamt 8096 Anrufe entgegen.

**Ergebnisse:**

Es wurden vor allem Depressions- (36 %), Angst- (18 %) und psychotische Symptome (19 %) berichtet. Dabei stand jeder zweite Anruf im Zusammenhang mit einer vorbestehenden psychischen Erkrankung. In den durchschnittlich 25-minütigen Beratungsgesprächen wurde eine Vielzahl psychologischer Akutinterventionen durchgeführt. Beim Vorliegen einer unklaren Symptomatik, psychotischer Symptomatik oder Symptomen schwerer Persönlichkeitsstörungen konnten die Berater*innen aus deren subjektiver Sicht signifikant weniger helfen als bei den restlichen Telefonaten, in denen klar umschriebene andere Symptome vorlagen.

**Diskussion:**

Die Ergebnisse weisen sowohl auf den Nutzen als auch die Grenzen von Hotlineangeboten hin. Der Nutzen besteht vor allem in der schnellen Verfügbarkeit sowie einer effektiven professionellen Hilfe bei klar charakterisierter Symptomatik. Bei unklarer oder komplexer Symptomatik scheint eine unmittelbare telefonische Hilfe zwar nur eingeschränkt möglich zu sein, sie kann den Zugang zu weiteren Hilfsangeboten jedoch einleiten. Insgesamt geben die Ergebnisse dieser Studie einen ersten Hinweis darauf, dass Hotlineangebote eine praktikable Möglichkeit zur psychologischen Ersthilfe unter Pandemiebedingungen darstellen.

## Abstract (en)

**Background:**

The COVID-19 pandemic represents a significant psychological burden for many people; however, especially during the first wave of the pandemic in Germany, little acute professional help was available for people in need.

**Objective:**

In southern Germany, a telephone hotline for psychological first aid for COVID-19-related burdens was set up under the lead of the Baden-Wuerttemberg Ministry of Social Affairs and Integration, opened to the entire population and evaluated in April 2020.

**Material and methods:**

In the period from 22 April to 24 July 2020, 753 volunteer psychotherapeutically trained counselors from different professional groups answered a total of 8096 calls.

**Results:**

Depression symptoms (36%), anxiety symptoms (18%) and psychotic symptoms (19%) were most frequently reported. Every second call was related to a previous mental illness. During the counseling sessions, which lasted 25 min on average, a variety of psychological acute interventions were conducted. In the presence of unclear symptoms, psychotic symptoms or severe personality disorder symptoms, the counselors were able to help significantly less compared to the remaining calls in which other clearly defined symptoms were present.

**Conclusion:**

The results point to both the benefits and limitations of hotline services. The major benefits relate to the fast availability and effective professional help for people with clearly characterized symptoms. In the case of unclear or complex symptoms, immediate help by telephone seems to be possible only to a limited extent, but it could initiate access to further help offers. Overall, the results of this study provide a first indication that hotline services for psychological first aid are feasible under pandemic conditions.

## Hintergrund

Die Covid-19-Pandemie stellt Deutschland und die gesamte Welt vor große medizinische, wirtschaftliche, soziale und auch psychologische Herausforderungen. Erste Längsschnittstudien deuten auf eine Zunahme der psychischen Belastung in der Bevölkerung hin [1, 2]. Unter einer Vielzahl pandemiebedingter Belastungsfaktoren stehen vor allem die Angst vor Infektionen, die staatlich verordneten Maßnahmen zur Eindämmung der Pandemie, wirtschaftliche Existenzängste und arbeitsbezogene Belastungen im Vordergrund.

Die Angst vor einer Infektion mit dem Coronavirus ist weit verbreitet. Demnach äußerten im März 2020, zum Höhepunkt der ersten Infektionswelle, 49 % aller Deutschen eine große oder sehr große Angst vor einer möglichen Infektion [3]. Eine deutsche Studie zeigte, dass die Corona-Angst zu Beginn der Pandemie schnell zunahm und mit einer späteren generalisierten Angst und Schlafstörungen einherging [4]. Die Angst zeigte sich besonders ausgeprägt bei vulnerablen Gruppen (z. B. Personen mit körperlichen Vorerkrankungen) oder aber bei Beschäftigten im Gesundheitswesen [5–7]. Dabei scheint es einen Zusammenhang zwischen der Angst vor einer Corona-Infektion mit psychischen Symptomen wie depressiven Beschwerden und suizidalen Gedanken sowie mit deutlichen psychosozialen Funktionseinschränkungen zu geben [8]. Neben der Angst, selbst durch das Coronavirus infiziert zu werden, bestehen ebenso Ängste, andere Personen anzustecken oder dass sich Personen im Freundes- und Familienkreis mit dem Virus infizieren könnten.

Die Eindämmung der Pandemie erforderte gerade während der ersten Welle zudem eine Serie von gänzlich neuen, staatlich verordneten Verhaltensempfehlungen und Präventionsmaßnahmen. Durch Isolations- und Quarantänemaßnahmen waren viele Menschen gezwungen, über lange Zeit alleine oder auf enge Räume beschränkt zu leben. Außerdem waren soziale Kontakte erschwert, wodurch wichtige Unterstützung entfiel. Die Auswirkungen dieser einschränkenden Maßnahmen auf die psychische Gesundheit von Menschen werden seit Beginn der Pandemie kritisch diskutiert. Schon im März 2020 erschien eine Übersichtsarbeit, in der traumaähnliche Symptome, Verwirrung und Ärger als mögliche Folgen von Quarantänemaßnahmen beschrieben wurden [9]. Die Belastungssymptome können auch noch Monate nach den Quarantänemaßnahmen bestehen bleiben [10]. Doch nicht nur die verordnete Quarantäne führt zu psychischen Belastungen, auch schon weniger extreme Formen der sozialen Distanzierung wie die Reduktion von Kontakten oder die Empfehlung, das häusliche Umfeld nicht zu verlassen, zeigte sich mit vermehrten Depressionssymptomen, generalisierten Angstsymptomen, intrusiven Gedanken, Insomnie und akutem Stress assoziiert [11]. Gerade ältere Menschen, aber auch Jugendliche, leiden demnach an den Folgen der Einschränkungen und berichten eine Zunahme von Einsamkeit und psychischer Belastung während der Pandemie [1, 12].

Nicht zuletzt stellen die volkswirtschaftlichen Folgen der Pandemie eine besondere Herausforderung dar. So führen Arbeitsplatzunsicherheit und finanzielle Sorgen im Rahmen der Corona-Pandemie zu Beeinträchtigungen der psychischen Gesundheit [13]. Homeoffice-Regelungen und Schulschließungen führen zu einer radikalen Veränderung des Lebensalltags vieler Menschen und stellen bisher ungekannte Herausforderungen dar, Arbeit, Kinderbetreuung und Haushalt zu vereinen. Eine Studie über die Auswirkungen von Homeschooling in sieben europäischen Ländern (u. a. Deutschland) zeigte negative Auswirkungen von Homeschooling sowohl für Eltern als auch für Kinder in Form von vermehrtem Stress, Sorgen, sozialer Isolation, häuslicher Konflikte und teilweise auch erhöhtem Alkoholkonsum [14]. Nicht zuletzt lösen die wirtschaftlichen Restriktionen für viele Menschen existenzielle Ängste aus [15], insbesondere bei Beschäftigten in besonders betroffenen Branchen wie der Gastronomie‑, Freizeit- oder Kulturbranche.

In vielen Fällen können diese Belastungen zu dysfunktionalen Bewältigungsversuchen führen wie erhöhtem Alkohol- und Substanzkonsum [16], Aggressivität und Wutausbrüchen oder ein Anstieg von Gewalt im häuslichen Umfeld [17–19]. In den europäischen Ländern wurde demnach im April 2020 ein Anstieg von Notrufen aufgrund häuslicher Gewalt gegen Frauen um 60 % im Vergleich zum Vorjahr verzeichnet [20]. Eine repräsentative Studie in Deutschland zeigte, dass die häusliche Gewalt gegen Frauen und Kinder unter Quarantänebedingungen um das zwei‑ bis dreifache zunahm [21].

Vor diesem Hintergrund besteht eine ernste Gefahr, dass psychische Erkrankungen durch die Pandemie zunehmen werden [22]. Die Bundespsychotherapeutenkammer warnt, dass die Corona-Pandemie manifeste psychische Erkrankungen auslösen oder verstärken werde, und benennt dabei v. a. Depressionen und Angststörungen, akute und posttraumatische Belastungsstörungen (PTBS), Alkohol- und Medikamentenabhängigkeit, Zwangsstörungen und Psychosen [23].

Es scheint daher von zentraler Bedeutung zu sein, Menschen bei der Bewältigung der psychischen Belastungen möglichst schnell, unkompliziert und früh zu unterstützen. Schon zu Beginn der Pandemie wurde daher auf die Wichtigkeit von Frühintervention hingewiesen [24]. Im Rahmen der Pandemie spielen dabei vor allem flexible und schnelle Hilfsangebote wie Online- oder Telefonhilfen eine wichtige Rolle, um die Menschen zu erreichen [25, 26]. Eine große Herausforderung besteht dabei, die Hotlinedienste mit ausreichend qualifizierten Berater*innen auszustatten [26].

In Deutschland konnten die gut etablierten Versorgungsstrukturen gerade zu Beginn der Pandemie nur bedingt greifen und pandemietaugliche Alternativkonzepte fehlten [27]. Daher gab es trotz der hohen Unsicherheit und subjektiv erlebten Bedrohung kaum Hilfsangebote, die eine unkomplizierte, schnelle und gleichzeitig therapeutisch professionelle Hilfe bei Corona-bedingten psychischen Belastungen sicherstellten. Aus Fachkreisen wurde daher darauf hingewiesen, dass eine Überlastung des bestehenden psychotherapeutischen und psychiatrischen Versorgungssystems, einschließlich der psychosozialen Dienste, wahrscheinlich sei und alternative Hilfsangebote wie Telefonhotlines dringend ausgebaut werden sollten [27, 28]. Aus dieser Situation heraus entstand das Vorhaben, die gegebenen psychotherapeutischen Versorgungsstrukturen zu nutzen, um ein professionelles, flexibles und niederschwelliges Angebot zur psychologischen Ersthilfe für die Allgemeinbevölkerung zu schaffen.

### Ziel des Projekts

Ziel des Projekts war es, Menschen mit psychischen Belastungen aufgrund der Corona-Pandemie mit einer schnellen, professionell ausgeübten Ersthilfe zu unterstützen. Dazu wurde sowohl eine Hotline eingerichtet, über die persönliche Beratungsgespräche durch Fachpersonen ermöglicht wurden, als auch eine Webseite, auf der Unterstützungsangebote zur psychologischen Selbsthilfe zusammengetragen wurden. Die Inanspruchnahme und Nutzung der Hilfsangebote wurde wissenschaftlich begleitet.

## Methodik

Das Hotlineprojekt wurde auf Initiative des Zentralinstituts für Seelische Gesundheit (ZI) und des Ministeriums für Soziales und Integration Baden-Württemberg in Zusammenarbeit mit der Landespsychotherapeutenkammer, der Landesärztekammer sowie der Kassenärztlichen Vereinigung Baden-Württemberg eingerichtet. Für die Hotline wurde eine zentrale Rufnummer des Landes Baden-Württemberg bereitgestellt. Die Hotline war zwischen dem 22.04. 2020 und dem 24.07.2020 täglich von 08:00 bis 20:00 Uhr erreichbar. Im Juli 2020 wurde die Hotline aufgrund der zwischenzeitlichen Entspannung der Corona-Situation und der nur noch marginalen Beteiligung der Berater*innen eingestellt.

### Rekrutierung der Berater*innen

Als potenzielle Berater*innen wurden durch die Landespsychotherapeutenkammer und die Landesärztekammer approbierte ärztliche und psychologische Psychotherapeut*innen sowie Kinder- und Jugendpsychotherapeut*innen per E‑Mail kontaktiert und um Beteiligung gebeten. Über staatlich anerkannte Ausbildungsinstitute wurden psychologische und Kinder- und Jugendpsychologische Psychotherapeut*innen in Ausbildung kontaktiert. Über das Sozialministerium wurden zudem erfahrene Sozialarbeiter*innen aus der gemeindepsychiatrischen Versorgung kontaktiert. Alle potenziellen Berater*innen erhielten ein Informationsschreiben, in dem sie über Ziel und Zweck der Hotline, den Ablauf der Registrierung und der Beratungen, der Freiwilligkeit ihrer Teilnahme sowie die bestehenden Datenschutzbestimmungen aufgeklärt wurden. Die Berater*innen konnten sich anschließend freiwillig über einen URL-Link registrieren. Nachdem die personale und berufliche Identität der Berater*innen geprüft wurde, wurden ihnen die Einwahldaten für die Hotline übersendet und ihre Telefonnummern zur Einwahl in die Hotline zugelassen. Die Berater*innen konnten sich anschließend zeitlich und örtlich flexibel mit ihrem eigenen Endgerät in die Hotline einwählen und so eingehende Anrufe überstellt bekommen. Über eine telefonische Direktschaltung mit der Kassenärztlichen Vereinigung war es den Berater*innen im Bedarfsfall möglich, den Anrufenden einen raschen Zugang zu psychotherapeutischer Behandlung zu ermöglichen. Insgesamt registrierten sich 753 Berater*innen, unter ihnen 469 ärztliche und psychologische sowie Kinder- und Jugendpsychotherapeut*innen, 107 Psychotherapeut*innen in Ausbildung, 160 Sozialarbeiter*innen im ambulant betreuten Wohnen und 17 Personen mit anderen psychosozialen Berufen.

### Implementierung der Webseite

Zusätzlich wurde eine Webseite mit Inhalten zur psychologischen Selbsthilfe eingerichtet. Betroffene konnten dort Tipps zu den folgenden Themen erhalten: „Psychische Krise & Gewalt“, „Tipps für den Alltag“, „Umgang mit Belastung und Stress“, „Sorgen und Ängste“, „Schlafprobleme“, „Mit sich alleine sein“, „Ärger & Konflikte“, „Familienleben“. Die Webseite ist unter der Adresse www.psyhotline-corona-bw.de weiterhin erreichbar.

### Datenerhebung

Das Projekt wurde durch Mitarbeiter*innen des ZI wissenschaftlich begleitet. Hierzu wurden Daten zur Inanspruchnahme der Hotline und zu den Beratungsgesprächen erhoben. Alle Datenerhebungen erfolgten vollständig anonym. Ein positives Ethikvotum der Ethikkommission II der Universität Heidelberg liegt vor (2020559N).

#### Dokumentation der Beratungen

**Figure 1:**
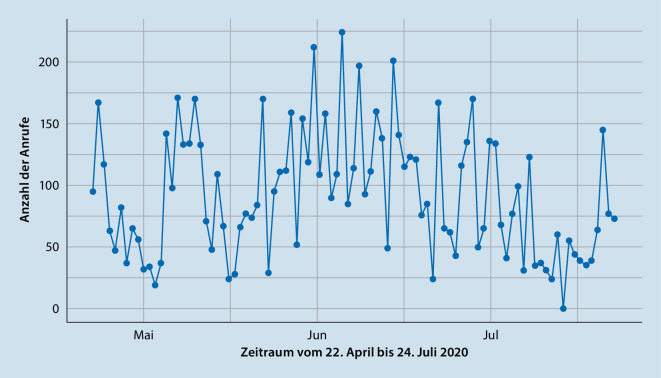

**Figure 2:**
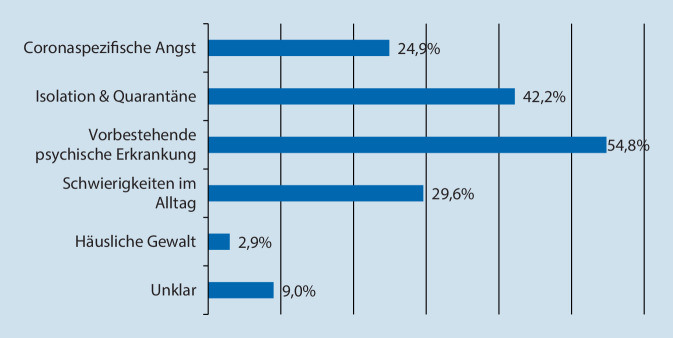

**Figure 3:**
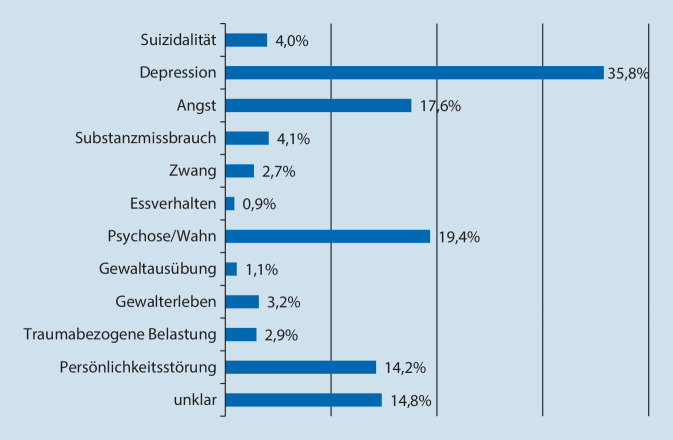

Die Anzahl der eingehenden Anrufe wurde vom Betreiber der Hotline zur wissenschaftlichen Auswertung bereitgestellt (Abb. 1). Zusätzlich wurden die Berater*innen gebeten, durchgeführte Beratungsgespräche über einen Onlinefragebogen zu dokumentieren. Im Fragebogen wurden Angaben zu Alter und Geschlecht der Anrufenden, dem Anrufgrund (Abb. 2), der aktuellen Belastungssymptomatik (Abb. 3) und den Inhalten der Beratungen erhoben. Bei allen Angaben waren Mehrfachnennungen möglich. Zusätzlich wurde die Gesprächsdauer sowie eine Einschätzung erhoben, wie sehr die Berater*innen den Anrufenden in den telefonischen Beratungen helfen konnten bzw. wie belastend sie diese empfunden haben (1 = überhaupt nicht, 5 = sehr viel).

#### Abschließende Berater*innenbefragung

In einer abschließenden Berater*innenbefragung wurde die Zufriedenheit der Berater*innen mit dem Projekt und ihrer Teilnahme erhoben.

### Statistische Analysen

Die Dokumentationen der Beratungen wurden deskriptiv ausgewertet. Die Einschätzungen der Berater*innen, den Anrufenden helfen zu können, wurde in Abhängigkeit der vorliegenden Symptomatik mithilfe von t‑Tests für unabhängige Stichproben inferenzstatistisch analysiert. Hierbei wurden die Angaben der Beratungsgespräche, in denen die jeweilige Symptomatik berichtet wurde, mit den Angaben der übrigen Beratungsgespräche verglichen, in denen die jeweilige Symptomatik nicht berichtet wurde (1 = liegt vor, 0 = liegt nicht vor). Um einen möglichen Zusammenhang zwischen der Inanspruchnahme der Hotline und dem Infektionsgeschehen in Deutschland zu analysieren, wurde die vom Robert-Koch-Institut veröffentlichte Anzahl der Neuinfektionen pro Tag in die Analysen mit einbezogen.

## Ergebnisse

Insgesamt war die Hotline über 13 Wochen vom 22.04. bis zum 24.07.2020 aktiv geschaltet. In diesem Zeitraum wurden insgesamt 8577 Anrufe registriert (Abb. 1). Die meisten Anrufe (27 %) gingen abends zwischen 18:00 und 20:00 Uhr ein. Es zeigte sich kein signifikanter Zusammenhang zwischen der Anzahl der eingehenden Anrufe und der Anzahl der Neuinfektionen pro Tag (*r* = −0,02, *p* = 0,983). Die Dauer eines Beratungsgesprächs betrug durchschnittlich 23,7 min (SD = 17,1). Insgesamt konnten 481 Anrufe (5,6 %) aufgrund eines vorübergehenden Beratermangels nicht durchgestellt werden, davon lassen sich 121 Anrufe auf technische Probleme an einem singulären Tag zurückführen. Die Anzahl der nicht durchgestellten Anrufe zeigte sich besonders hoch in den Morgenstunden zwischen 08:00 bis 09:00 Uhr (79 Anrufe), in den Mittagsstunden zwischen 12:00 bis 13:00 Uhr (77 Anrufe) und in den Abendstunden zwischen 18:00 bis 19:00 Uhr (95 Anrufe). Somit konnten insgesamt 8096 eingehende Anrufe durchgestellt werden.

### Dokumentation der Beratungsgespräche

Von den 8096 durchgestellten Anrufen wurden insgesamt 1292 Telefonberatungen dokumentiert (16 %). Demnach, waren 64 % der Anrufenden weiblich und die große Mehrheit der Anrufenden erwachsen (79 %), gefolgt von Anrufenden höheren Alters (17 %). Nur ein kleiner Teil der Anrufenden waren Jugendliche (3,8 %) oder Kinder (0,2 %). Mehr als jeder zweite dokumentierte Anruf stand in Zusammenhang mit einer vorbestehenden psychischen Erkrankung (55 %), gefolgt von Anrufen aufgrund von Quarantänemaßnahmen oder sozialer Isolation (42 %), Problemen im Alltag (30 %) oder Corona-spezifischen Ängsten (25 %). Nur ein kleiner Teil der Anrufe bezog sich auf häusliche Gewalt (3 %; Abb. 2).

Die mit Abstand am häufigsten dokumentierte Symptomatik waren depressive Symptome (36 %), gefolgt von psychotischen Symptomen (19 %) und Angstsymptomen (18 %). 15 % der dokumentierten Anrufe berichteten eine unklare Symptomatik. Nur ein kleiner Teil (4 %) berichtete Suizidgedanken oder -absichten (Abb. 3). Die durchschnittliche Belastung der Anrufenden lag bei M = 3,3 (SD = 0,8) auf einer Skala von 1 bis 5.

In 42 % aller Beratungen gaben die Berater*innen an, kurze therapeutische Interventionen durchgeführt zu haben. Diese umfassten vor allem unterstützende Gesprächstechniken (z. B. Validierung, supportive Techniken), Anleitung zu Entspannung (z. B. Atemübungen), emotionsfokussierte Interventionen (z. B. Benennen und Sortieren von Gefühlen, entgegengesetztes Handeln), kognitive Interventionen (z. B. hilfreiche Sätze, Perspektivwechsel, Grübelmanagement), verhaltensbezogene Interventionen (z. B. Tagesstruktur, positive Aktivitäten), psychoedukative Interventionen (z. B. Schlafhygiene), Unterstützung bei Problemlösung (z. B. Konfliktberatung) sowie Ressourcenaktivierung. In 26 % aller dokumentierten Beratungen wurde eine psychotherapeutische Behandlung empfohlen, in 11 % aller Beratungen auf weitere, spezialisierte Telefonangebote verwiesen. In 16 % der Beratungen wurden Informationen zu Corona vermittelt. Nur in wenigen Beratungen (2 %) war ein akutes Krisencoaching notwendig (z. B. aufgrund akuter Suizidalität).

Insgesamt gaben die Berater*innen an, dass sie in 27 % aller Gespräche „(sehr) viel“, in 40 % „etwas“ und in 33 % „nur wenig“ oder „gar nicht“ haben helfen können. Dabei zeigte sich, dass die Berater*innen in Beratungsgesprächen, bei denen ein klarer Anrufgrund vorlag (z. B. Corona-Angst, Isolation und Quarantäne, Schwierigkeit im Alltag oder Gewalt), signifikant mehr helfen konnten, im Vergleich zu den übrigen Beratungsgesprächen, in denen diese Anrufgründe nicht vorlagen (Tab. 1). In Beratungsgesprächen, in denen der Anrufgrund unklar war, konnten die Berater*innen im Vergleich zu den übrigen Beratungsgesprächen signifikant weniger helfen (M = 1,84). Ein ähnliches Bild zeigte sich auf der Symptomebene. Beim Vorliegen einer klaren Symptomatik wie Depressions‑, Angst‑, Zwangssymptomen, Gewalterleben oder PTBS konnten die Berater*innen signifikant mehr helfen (M = 2,98–3,34) als bei den übrigen Beratungen, in denen die jeweilige Symptomatik nicht vorlag. Signifikant weniger helfen konnten die Berater*innen hingegen im Vergleich zu den übrigen Beratungsgesprächen beim Vorliegen von psychotischen Symptomen, Störungen auf Persönlichkeitsebene oder bei unklarer Symptomatik (M = 2,31–2,50). Keine Unterschiede zeigten sich zwischen Beratungsgesprächen, in denen akute Suizidalität, Essstörungen oder Gewaltausübungen vorlagen (M = 2,71–3,27) im Vergleich zu den übrigen Beratungsgesprächen.

**Table 1:** 

	Grund/Symptom liegt vor			Grund/Symptom liegt nicht vor			*t*-Test			Effekt
	M	SD	*n*	M	SD	*n*	*t*-Wert	Df	*p*-Wert	d
*Grund für Anruf*										
Corona-Angst	3,30	0,87	315	2,67	1,06	900	10,32	664	<0,001	0,61
Quarantäne	3,06	0,94	535	2,65	1,10	680	6,93	1205	<0,001	0,39
Psych. Erkrankung	2,79	1,02	692	2,89	1,10	523	−1,71	1077	0,087	−0,10
Schwierigkeit Alltag	2,97	0,97	369	2,77	1,08	846	3,08	774	0,002	0,18
Häusliche Gewalt	3,24	0,86	37	2,82	1,05	1178	2,92	39	0,006	0,40
Unklar	1,84	1,02	94	2,92	1,01	1121	−9,83	109	<0,001	−1,06
*Symptome*										
Suizidalität	2,71	0,94	49	2,84	1,06	1166	−0,90	53	0,372	−0,12
Depression	2,98	0,94	456	2,74	1,10	759	4,02	1078	<0,001	0,23
Angst	3,32	0,79	221	2,72	1,07	994	9,49	423	<0,001	0,58
Zwang	3,18	0,90	34	2,82	1,05	1181	2,24	36	0,032	0,34
Essverhalten	3,27	0,90	11	2,83	1,05	1204	1,62	10	0,136	0,42
Psychose/Wahn	2,42	1,03	243	2,94	1,03	972	−6,93	374	<0,001	−0,50
Gewaltausübung	2,93	0,83	14	2,83	1,05	1201	0,43	14	0,672	0,09
Gewalterleben	3,33	0,89	40	2,82	1,05	1175	3,54	43	<0,001	0,49
Posttraumatische Belastungsstörungen	3,34	0,89	38	2,82	1,05	1177	3,61	41	<0,001	0,5
Persönlichkeit	2,50	1,00	181	2,89	1,05	1034	−4,76	254	<0,001	−0,37
Unklar	2,31	1,06	175	2,92	1,02	1040	−7,11	232	<0,001	−0,60

Restkategorie „weiß nicht“ (*k* = 31); keine Angabe (k = 46)

*M* Mittelwert, *SD* Standardabweichung, *n* Anzahl, *d* Standardisierte Mittelwertdifferenz (Cohen’s d), *Df* Freiheitsgrade

Mit einem Mittelwert von 1,9 (SD = 0,9) auf einer Skala von 1 (gar nicht) bis 5 (sehr), empfanden die Berater*innen die Gespräche im Durchschnitt nur wenig belastend. Nur 5 % der Gespräche wurden als (sehr) stark belastend empfunden.

### Inanspruchnahme der Webseite

**Figure 4:**
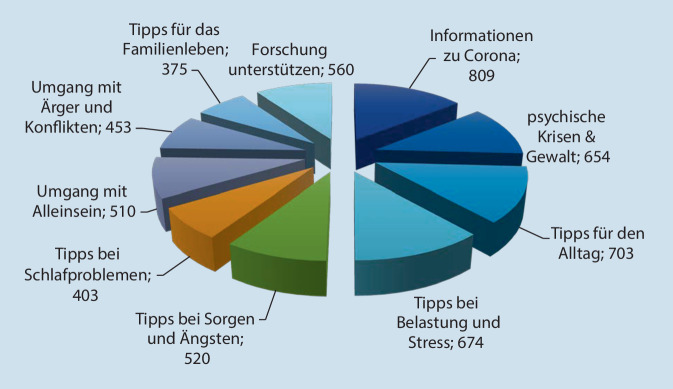

Die begleitende Webseite verzeichnete im selben Zeitraum, zwischen März und Juli 2020, insgesamt 5544 Aufrufe, also durchschnittlich 656 Aufrufe pro Tag (Abb. 4). Die Aufrufe auf der Webseite verteilten sich gleichmäßig über alle Rubriken hinweg.

### Abschließende Berater*innenbefragung

In der abschließenden Berater*innenbefragung zeigte sich eine hohe Zufriedenheit der Berater*innen mit dem Projekt. Auf einer 5‑stufigen Skala (1 = überhaupt nicht; 5 = sehr) bewerteten die Berater*innen das Hotlineprojekt durchschnittlich als sinnvoll (M = 4,03, SD = 1,06), die zugehörige Webseite als hilfreich (M = 4,02, SD = 1,04) und die eigene Beteiligung als teilweise befriedigend (M = 3,34, SD = 1,04).

## Diskussion

Ziel des Projekts war es, eine schnelle und professionelle psychologische Ersthilfe für Menschen mit psychischen Belastungen in der ersten Welle der Corona-Pandemie zu ermöglichen. Dazu wurden eine Hotline zur persönlichen Beratung und eine Webseite für Selbsthilfezwecke eingerichtet.

Mit 753 registrierten Berater*innen stieß das Hotlineprojekt auf eine große berufsgruppenübergreifende Zustimmung. Durch die hohe Anzahl professioneller Berater*innen konnte eine hohe Beratungsqualität sichergestellt werden, was in bisherigen Hotlineimplementierungen eine große Herausforderung darstellte [26].

Mit insgesamt 8577 Anrufen in 13 Wochen (ca. 700 Anrufe pro Woche) stieß die Hotline auf eine große Nachfrage in der Landesbevölkerung. Die Häufung der Anrufe in den Abendstunden zeigt die Wichtigkeit, diese Hilfsangebote auch außerhalb der regelhaften Arbeitszeit anzubieten [26]. Die Anzahl der eingehenden Anrufe zeigte keinen Zusammenhang mit der Anzahl der Neuinfektionen pro Tag. Dies könnte darauf zurückzuführen sein, dass die Hotline erst Ende April, kurz nach dem Höhepunkt der ersten Welle, in Betrieb genommen wurde. Die Anzahl der Neuinfektionen nahm zu diesem Zeitpunkt bereits kontinuierlich ab, während die Beschränkungsmaßnahmen nur langsam gelockert wurden. Wahrscheinlich ist auch, dass weitere Faktoren, wie die Bekanntheit der Hotline, einen wichtigen Einfluss auf die Anrufzahlen hatten.

Die Dokumentationen der Berater*innen weisen darauf hin, dass jeder zweite Anruf von Personen getätigt wurde, die eine bereits erkannte psychische Störung angaben. Dies könnte mit besonderen Risiken dieser Personengruppe zusammenhängen, was sich mit Erkenntnissen aus weiteren Studien deckt. So zeigte sich, dass COVID-19-erkrankte Personen mit komorbiden psychischen Störungen im Vergleich zu COVID-19-Erkrankten ohne psychische Störung eine um 48 % erhöhte Mortalitätsrate aufweisen [29]. Diese Befunde unterstreichen die Wichtigkeit, dieser gefährdeten Gruppe besondere Aufmerksamkeit zu schenken [30]. Wie erwartet betrafen die weiteren Gründe für einen Anruf bei der Hotline vor allem Corona-spezifische Ängste, Belastungen aufgrund der Isolations- und Quarantänemaßnahmen oder Schwierigkeiten im Alltag (25–42 %). Der Anteil der Anrufe aufgrund häuslicher Gewalt lag mit 2,9 % vergleichsweise niedrig, obwohl diese auch in Deutschland unter Quarantänemaßnahmen signifikant zugenommen hat [21]. Dies kann auf mehrere Gründe zurückzuführen sein: Zum einen gibt es weitere etablierte und gut ausgebaute telefonische Hilfsangebote für diese spezifische Zielgruppe, zum anderen jedoch sind Hilfsangebote bei häuslicher Gewalt den Betroffenen in vielen Fällen nicht bekannt oder es ist ihnen nur eingeschränkt möglich, bei weitreichender Überwachung und Kontrolle durch eine Partner*in, diese telefonischen Hilfsangebote zu nutzen [21]. Dies legt nahe, dass Hilfsangebote in der Öffentlichkeit besser kommuniziert und unterschiedliche Zugänge zu diesen Angeboten geschaffen werden sollten (z. B. Online und Telefon).

Auf Symptomebene zeigten sich hohe Anteile von Depressions- und Angstsymptomen sowie ein überraschend hoher Anteil psychotischer und wahnhafter Symptome. Möglicherweise trägt das niedrigschwellig und anonym erreichbare Angebot dazu bei, dass Psychosebetroffene ein psychotherapeutisches Beratungsangebot annehmen. Der hohe Anteil dieser Symptomgruppen deckt sich mit der Einschätzung der Bundespsychotherapeutenkammer, dass Depressionen, Angststörungen und Psychosen zu den psychischen Erkrankungen gehören, die besonders durch die Pandemie ausgelöst oder verstärkt werden können [23]. Der hohe Anteil von Depressions- und Angstsymptomen zeigt sich in Übereinstimmung mit der vergleichsweise hohen Prävalenz dieser Erkrankungen in der deutschen Allgemeinbevölkerung mit 9,3 % für eine affektive Störung und 15,3 % für eine Angststörung [31], während der hohe Anteil psychotischer Symptome den hohen Bedarf an psychotherapeutischen Hilfen für diese Personengruppe anzeigt. Symptome von Zwangsstörungen, posttraumatischer Belastungsstörung oder Substanzkonsum wurden hingegen mit 3–4 % vergleichsweise seltener berichtet.

Bemerkenswert erscheint die Vielzahl an Kurzinterventionen, die trotz der anonymen Anrufe akut telefonisch durchgeführt werden konnten. Diese umfassten unterstützende Gesprächstechniken, Anleitung zu Entspannung, emotionsfokussierte, kognitive und verhaltensbezogene Interventionen, psychoedukative Interventionen, Unterstützung bei Problemlösung sowie Ressourcenaktivierung. An dieser Stelle sei noch einmal die Relevanz psychotherapeutisch geschulter Berater*innen herausgestellt, die auch in kurzer Zeit im telefonischen Kontakt gezielt helfen können.

Die Einschätzung der Berater*innen, wie sehr sie den Anrufenden haben helfen können, zeigt, dass diese bei etwa zwei Drittel aller Beratungsgespräche den Eindruck hatten, zumindest „etwas“, häufig aber auch „viel“ oder „sehr viel“ helfen zu können. Dabei konnten die Berater*innen vor allem dann helfen, wenn ein klarer Anrufgrund oder eine klare Symptomatik vorlag. Bei unklaren Anrufgründen und unklarer Symptomatik konnten die Berater*innen signifikant weniger helfen; insbesondere bei psychotischen Symptomen und Symptomen von Persönlichkeitsstörungen gaben die Berater*innen an, dass sie weniger helfen konnten. Dennoch ist denkbar, dass auch dieser Personengruppe ein Anstoß gegeben wurde, sich weiterhin um psychotherapeutische Hilfen zu bemühen.

### Limitationen

Zur Gewährleistung der Anonymität und des Datenschutzes wurden die Anrufenden nicht direkt um Angaben zur Person oder zu den Beratungsgesprächen gebeten. Bei der Datenerhebung handelt es sich lediglich um freiwillige Angaben der Berater*innen. Aufgrund der Freiwilligkeit der Dokumentation wurden zudem lediglich 16 % der Beratungsgespräche dokumentiert (1292 von 8096 durchgestellten Anrufen). Die Ergebnisse sollten daher mit Vorsicht und die vorliegenden Symptome nicht als Feststellung einer gesicherten Diagnose interpretiert werden. Obwohl die Dokumentationen von Fachpersonal durchgeführt wurden, die langjährige Erfahrungen bei der Einschätzung psychischer Symptome und deren Behandlung aufweisen, stellen die Ergebnisse nur einen ersten Hinweis auf den Nutzen einer entsprechenden Hotline dar. Diesem sollte durch zusätzliche Befragungen der Anrufenden in Zukunft weiter nachgegangen werden.

### Schlussfolgerung

Insgesamt leistete die psychologische Hotline einen wichtigen Beitrag zur Bewältigung psychischer Belastungen während der ersten Infektionswelle der Corona-Pandemie in Baden-Württemberg. Für viele Menschen, die in diesem Zusammenhang unter psychischen Belastungen litten, wurde so eine Möglichkeit zur schnellen und professionellen psychologischen Ersthilfe geschaffen. Ein besonderer Dank gebührt der Vielzahl ehrenamtlicher Berater*innen, die sich über verschiedene Berufsgruppen und Therapieschulen hinweg engagierten, um einen außerordentlich wichtigen gesellschaftlichen Beitrag zur Bewältigung der Pandemie zu leisten.

## Fazit für die Praxis

Die Ergebnisse dieser Arbeit zeigen sowohl den Nutzen als auch die Grenzen von Hotlineangeboten auf. Der Nutzen besteht in erster Linie in der schnellen und einfachen Verfügbarkeit einer psychologischen Ersthilfemaßnahme. Bei unklarer oder komplexer psychischer Symptomatik scheint eine direkte telefonische Hilfe zwar nur eingeschränkt möglich zu sein, sie kann den Zugang zu einem fachärztlichen oder fachpsychotherapeutischen Kontakt zur Bewältigung der Belastungen jedoch erleichtern. Einen Ersatz für persönliche Kontakte sollte ein solches Hotlineangebot aber unter keinen Umständen darstellen. Insgesamt geben die hohe Nachfrage in der Bevölkerung sowie die Angaben zu Inhalt und Nutzen der Beratungsgespräche durch die Berater*innen einen ersten Hinweis darauf, dass Hotlineangebote eine praktikable Möglichkeit zur psychologischen Ersthilfe unter Pandemiebedingungen darstellen.

## References

[1] Daly M, Sutin A, Robinson E (2020). Longitudinal changes in mental health and the COVID-19 pandemic: evidence from the UK Household Longitudinal Study. Psychol Med.

[2] Pierce M, Hope H, Ford T (2020). Mental health before and during the COVID-19 pandemic: a longitudinal probability sample survey of the UK population. Lancet Psychiatry.

[3] Statista (2020). Gefühle in Bezug auf eine Ansteckung mit dem Corona Virus.

[4] Hetkamp M, Schweda A, Bäuerle A (2020). Sleep disturbances, fear, and generalized anxiety during the COVID-19 shut down phase in Germany: relation to infection rates, deaths, and German stock index DAX. Sleep Med.

[5] Shevlin M, Mcbride O, Murphy J (2020). Anxiety, depression, traumatic stress, and COVID-19 related Anxiety in the UK general population during the COVID-19 pandemic.

[6] Bohlken J, Schömig F, Lemke MR (2020). COVID-19-Pandemie: Belastungen des medizinischen Personals: Ein kurzer aktueller review. Psychiatr Prax.

[7] Kontoangelos K, Economou M, Papageorgiou C (2020). Mental health effects of COVID-19 pandemia: a review of clinical and psychological traits. Psychiatry Investig.

[8] Lee SA, Jobe MC, Mathis AA (2020). Mental health characteristics associated with dysfunctional coronavirus anxiety. Psychol Med.

[9] Brooks SK, Webster RK, Smith LE (2020). The psychological impact of quarantine and how to reduce it: rapid review of the evidence. Lancet.

[10] De Lima CVC, Cândido EL, Da Silva JA (2020). Effects of quarantine on mental health of populations affected by Covid-19. J Affect Disord.

[11] Marroquín B, Vine V, Morgan R (2020). Mental health during the COVID-19 pandemic: Effects of stay-at-home policies, social distancing behavior, and social resources. Psychiatry Res.

[12] Luchetti M, Lee JH, Aschwanden D (2020). The trajectory of loneliness in response to COVID-19. Am Psychol.

[13] Wilson JM, Lee J, Fitzgerald HN (2020). Job insecurity and financial concern during the COVID-19 pandemic are associated with worse mental health. J Occup Environ Med.

[14] Thorell L, Skoglund CB, De La Peña AG (2020). Psychosocial effects of homeschooling during the COVID-19 pandemic: differences between seven European countries and between children with and without mental health conditions.

[15] Rigotti T, De Cuyper N, Sekiguchi T (2020). The corona crisis: what Can we learn from earlier studies in applied psychology? J. Appl Psychol.

[16] Rodriguez LM, Litt DM, Stewart SH (2020). Drinking to cope with the pandemic: the unique associations of COVID-19-related perceived threat and psychological distress to drinking behaviors in American men and women. Addict Behav.

[17] Chandan JS, Taylor J, Bradbury-Jones C (2020). COVID-19: a public health approach to manage domestic violence is needed. Lancet Public Health.

[18] Mazza M, Marano G, Lai C (2020). Danger in danger: Interpersonal violence during COVID-19 quarantine. Psychiatry Res.

[19] Xue J, Chen J, Chen C (2020). The hidden pandemic of family violence during COVID-19: unsupervised learning of tweets. J Med Internet Res.

[20] Mahase E (2020). Covid-19: EU states report 60 % rise in emergency calls about domestic violence. BMJ.

[21] Steinert J, Ebert C (2020) Gewalt an Frauen und Kindern in Deutschland während COVID-19-bedingten Ausgangsbeschränkungen: Zusammenfassung der Ergebnisse. Unpubliziertes Manuskript, Technische Universität München

[22] Pfefferbaum B, North CS (2020). Mental health and the Covid-19 pandemic. N Engl J Med.

[23] Bptk (2020) Corona-Pandemie und psychische Erkrankungen. BPtK-Hintergrund zur Forschungslage. https://www.bptk.de/wp-content/uploads/2020/08/2020-08-17_BPtK-Hintergrund_Corona-Pandemie-und-psychische-Erkrankungen.pdf. Zugegriffen: 3. Nov. 2020

[24] Galea S, Merchant RM, Lurie N (2020). The mental health consequences of COVID-19 and physical distancing: the need for prevention and early intervention. JAMA Intern Med.

[25] Liu S, Yang L, Zhang C (2020). Online mental health services in China during the COVID-19 outbreak. Lancet Psychiatry.

[26] Wang J, Wei H, Zhou L (2020). Hotline services in China during COVID-19 pandemic. J Affect Disord.

[27] Zielasek J, Gouzoulis-Mayfrank E (2020). COVID-19-Pandemie: Psychische Störungen werden zunehmen. Dtsch Arztebl.

[28] Brakemeier EL, Wirkner J, Knaevelsrud C, Wurm S, Christiansen H, Lueken U, Schneider S (2020). Die COVID-19-Pandemie als Herausforderung für die psychische Gesundheit. Z Klin Psychol Psychother.

[29] Wang Q, Xu R, Volkow ND (2021). Increased risk of COVID-19 infection and mortality in people with mental disorders: analysis from electronic health records in the United States. World Psychiatry.

[30] Yao H, Chen J-H, Xu Y-F (2020). Patients with mental health disorders in the COVID-19 epidemic. Lancet Psychiatry.

[31] Jacobi F, Höfler M, Siegert J (2014). Twelve-month prevalence, comorbidity and correlates of mental disorders in Germany: the Mental Health Module of the German Health Interview and Examination Survey for Adults. Int J Methods Psychiatr Res.

